# Detection of Genes Associated with Polymyxin and Antimicrobial Peptide Resistance in Isolates of *Pseudomonas aeruginosa*

**DOI:** 10.3390/ijms262110499

**Published:** 2025-10-29

**Authors:** Meseret Alem Damtie, Ajay Kumar Vijay, Mark Duncan Perry Willcox

**Affiliations:** 1 School of Optometry and Vision Science, University of New South Wales, Sydney, NSW 2052, Australia; m.damtie@unsw.edu.au (M.A.D.); ajay.vijay@wimr.org.au (A.K.V.); 2 Centre for Vision Research, Westmead Institute for Medical Research, The University of Sydney, Sydney, NSW 2145, Australia

**Keywords:** polymyxin, *Pseudomonas aeruginosa*, antimicrobial peptides, antimicrobial resistance

## Abstract

*Pseudomonas aeruginosa* causes ocular and other infections and quickly acquires antimicrobial resistance. Polymyxin B and colistin are last-line agents against resistant *P. aeruginosa*, yet even resistance to these is increasing. Antimicrobial peptides (AMPs) are also being developed as new antibiotics, but resistant mechanisms to polymyxins might also cause resistance to these AMPs. This study evaluated whether isolates with differing polymyxin resistances also showed elevated minimum inhibitory concentrations (MICs) to the human cathelicidin LL-37 and a synthetic AMP, Mel4. Forty isolates of *P. aeruginosa,* mostly collected in India and Australia, were assessed for minimum inhibitory concentrations (MICs) by broth microdilution in cation-adjusted Mueller–Hinton broth. Whole genome sequences were analyzed using NCBI BLAST (version 2.17.0). SNPs vs. MIC associations were evaluated with Fisher’s exact test. Sixty-five percent of isolates were resistant to polymyxin B, and 80% to colistin. Polymyxin B MICs ranged from 0.5 to 512 µg/mL, with 32.5% showing intermediate resistance and 22.5% being highly resistant (MIC ≥ 256 µg/mL). MICs for polymyxin B and colistin were strongly correlated with each other (Spearman’s R ≥ 0.6; n = 40; *p* ≤ 0.001). LL-37 showed moderate correlations with polymyxin B, colistin, and Mel4, whereas Mel4 showed weaker correlations with polymyxin B or colistin (R < 0.4). Genomic analysis identified SNPs in *mipB* (V469M, G441S) as being associated with the MICs to all the antimicrobials. Strains with MICs between 64 and 512 µg/mL were significantly more likely to harbor *nalC* (E153Q/D) or the *mipB* variants (*p* < 0.05). Higher polymyxin MICs were associated with elevated MICs to LL-37 and, to a lesser extent, Mel4, suggesting partial shared resistance among membrane active peptides. Defining the effect of the SNPs and clinical relevance of AMP cross-resistance may inform future therapies and safer contact lenses.

## 1. Introduction

Antimicrobial resistance has emerged as a pressing global health challenge, with approximately 700,000 deaths each year attributed to resistant microbes. This number is projected to reach a staggering 10 million deaths annually, accompanied by economic losses of $100 trillion, by 2025 [1]. Recognizing its global threat, both the Centers for Disease Control and Prevention and the World Health Organization have classified *P. aeruginosa* as a priority pathogen, highlighting the urgent need for novel antimicrobial strategies.

*Pseudomonas aeruginosa* is a Gram-negative, opportunistic pathogen and a major cause of numerous kinds of infections. It poses a significant threat, particularly to immunocompromised individuals, including those with cystic fibrosis, burn injuries, cancer, or prolonged hospital stays [2,3]. It can cause pneumonia, bloodstream infections, urinary tract infections, and surgical site infections [4]. It is also the leading cause of sight-threatening corneal disease in otherwise healthy patients who use contact lenses [5,6,7,8].

Its ability to rapidly develop antimicrobial resistance makes it a challenging pathogen in both ophthalmic and systemic infections, necessitating effective surveillance and treatment strategies [9]. It can evade antimicrobial therapy through multiple resistance mechanisms. These include active efflux systems such as the MexAB-OprM efflux pump, enzymatic degradation (e.g., β-lactamases), and modifications of the outer membrane that reduce antibiotic permeability. In recent years, there has been an increasing prevalence and dissemination of multidrug-resistant (MDR) and extensively drug-resistant (XDR) *P. aeruginosa*, with rates of between 15 and 30% in some geographical areas [10,11,12]. Its high adaptability and extensive resistance means infections caused by *P. aeruginosa* are associated with prolonged hospital stays, increased morbidity, high mortality rates, and increased cost of healthcare [11,13].

Due to the shortage of novel antimicrobials, the lipopeptide antibiotics polymyxin B and colistin (polymyxin E) are often used as last-resort antibiotics to treat MDR/XDR *P. aeruginosa* infections [14]. These cationic cyclic lipopeptides disrupt the bacterial outer membrane by binding to lipopolysaccharides (LPS) and displacing divalent cations (Ca^2+^ and Mg^2+^) from the phosphate groups, leading to membrane destabilization and cell death [3]. Also, there is development of antimicrobial peptides (AMP) as potential new antibiotics. These have similarities to polymyxins, being generally cationic and amphiphilic and reacting with the membranes of bacteria as their most common mode of action [15,16]. The naturally occurring AMP LL-37, the only human cathelicidin, has probably received the most attention for development, although it has yet to reach the clinic [17].

Unfortunately, there is emergence of polymyxin resistance in *P. aeruginosa* around the world [14]. This resistance occurs most commonly as a result of modification in the lipid A moiety of LPS [18], as well as from efflux of the drugs via pumps. These mechanisms can act synergistically to enhance drug resistance. LPS of *P. aeruginosa* consists of three distinct domains, a core oligosaccharide, the lipid A portion and the O-antigen region [19]. Modifications of lipid A reduce the electrostatic binding affinity of polymyxins and occur during exposure to polymyxins. Chemically, it is the incorporation of 4-amino-4-deoxy-L-arabinose (L-Ara4N), phosphoethanolamine, and galactosamine into lipid A that reduces its negative charge and consequently the affinity for binding with positively charged polymyxins [20]. This LPS modification in *P. aeruginosa* is regulated by several two-component signaling systems, PmrAB (also known as BasRS), PhoPQ, CprRS, ParRS, and ColRS. These systems activate overlapping resistance pathways, particularly the *arnBCADTEF* operon, which leads to the addition of L-Ara4N to lipid A [21], ensuring a robust resistance response even if one pathway is disrupted (not functional) [22,23].

Efflux pumps play a secondary but significant role in polymyxin resistance by reducing intracellular drug accumulation. The MexAB-OprM efflux pump, a major resistance-nodulation-cell division (RND) transporter in *P. aeruginosa*, actively expels a broad range of antibiotics, including polymyxins [24,25]. Under normal conditions, MexR represses *mexAB-oprM* expression. However, mutations in *mexR* disrupt its function, leading to efflux pump overexpression and increased antibiotic efflux [25]. Regulatory proteins such as CpxR also contribute to resistance by modulating stress adaptation genes, which subsequently enhance efflux pump expression [26]. A polymyxin-induced operon, *mipAB*, can be activated by the ParRS two-component regulatory system, and these act in concert to activate the synthesis of the MexXY-OprA efflux pump [27].

AMPs form an important part of the human defense system in many body sites and have been shown to be important in controlling ocular infections. The mouse cathelicidin CRAMP (its human LL-37 homolog) as well as β-defensins-2 and 3 protect the eye against *P. aeruginosa* infections [28,29,30]. Whilst bacteria struggle to develop resistance to AMPs in the laboratory in experiments where they are exposed to increasing concentrations of AMPs [31], resistance mechanisms are known, and these can overlap with mechanisms that bacteria use to become resistant to polymyxins. This includes lipid A modification with 4-amino-L-arabinose and active efflux from cells [32]. This implies that resistance to polymyxins may confer resistance to AMPs, even those of the innate human defense system such as LL-37. If this is the case, this may make polymyxin-resistant strains much more virulent as they could also evade the AMP-based defense system.

The aim of this study was to examine strains of *P. aeruginosa* for their susceptibility to polymyxin antibiotics and the AMPs LL-37 and Mel4, and to examine whether *P. aeruginosa* ocular isolates with different minimum inhibitory concentrations (MICs) to polymyxins also showed higher MICs with LL-37 and a synthetic AMP Mel4 which was used as an antimicrobial coating for contact lenses in a Phase III clinical trial [33]. Furthermore, whole genome sequences of the strains were analyzed to identify known polymyxin resistance genes and single nucleotide polymorphisms (SNPs) within these genes and to assess their associations with elevated MICs to polymyxins or AMPs.

## 2. Results

### 2.1. Antimicrobial Susceptibility to the Peptide Antibiotics

The MICs of polymyxin B, colistin, Mel4, and LL-37 against *P. aeruginosa* are shown in Table 1. For polymyxin and colistin, the Clinical and Laboratory Standards Institute defines an intermediate break point at (MIC ≤ 2 µg/mL) and a resistant breakpoint of (MIC ≥ 4 µg/mL). No susceptible breakpoint has been established. Among the 40 *P. aeruginosa* clinical and reference isolates tested, 65% were resistant to polymyxin B and 80% to colistin. The MIC of polymyxin B ranged from 0.5 to 512 µg/mL, with the geometric mean MIC being 19.0 µg/mL, while the median MIC was 40.0 µg/mL. A total of 13 strains showed intermediate resistance to polymyxin B (32.5% of strains) and 9 isolates had very high levels of resistance (MIC ≥ 256 µg/mL; 22.5% of strains). The MIC values for colistin ranged from 0.5 to 512 µg/mL, with the geometric mean MIC being 20.4 µg/mL and the median being 16.0 µg/mL. A total of eight strains showed intermediate resistance (20%) and six strains (15%) had very high levels of resistance (MIC ≥ 256 µg/mL). Mel4 had a geometric mean MIC of 50.2 µg/mL, with a median of 48 µg/mL. The MIC ranged between 0.5 and 256 µg/mL. For LL-37, the geometric mean MIC was 51.1 µg/mL, with a median of 48 µg/mL and range of 0.5 to 256 µg/mL.

There was a strong ($r_{s}$ ≥ 0.6) positive correlation between polymyxin and colistin MIC values (Table 2); this suggests clinical isolates that are resistant to polymyxin were likely to also be resistant to colistin. There were moderate positive correlations ($r_{s}$ ≥ 0.4) between the MIC for LL-37 and those for polymyxin B, colistin, and Mel4. For Mel4, there was a weak ($r_{s}$ < 0.4) positive correlation with MICs for polymyxin B and colistin (Table 2).

The correlations between MICs to the four AMPs indicated that there was the possibility of shared genetic determinants of resistance.

### 2.2. Possession of Genes and SNPs Associated with Polymxin Resistance

Table 3 gives the data for genes involved in polymyxin resistance associated with the *arnBCADTEF* operon and its regulation (*pmrAB*, *phoPQ*, *cprRS*, *parRS*, and *colRS*). All strains possessed every gene, but strains were distinct in the possession of single nucleotide polymorphisms (SNPs) within these genes. For example, strains PA189, PA182, PA224, PA206, PA126, PA193, PA225, PA198, ATCC 19660, PA216, PA220, and PA123 possessed the SNP that led to the L71R amino acid change in PmrA, whereas strains PA33, PA223, PA31, 6206, PA221, PA55, PA217, and PA219 did not possess any SNPs in this gene. Also, strains PA206, PA193, PA225, and PA198 possessed the SNP leading to the amino acid change D61E, strain PA126 possessed the SNP leading to V34L, and strain PA227 possessed the SNP leading to the T31I amino acid change in PmrA. Table 4 gives data for genes involved in efflux pumps and their regulation (*mipA*, *mipB*, *armR*, *mexR*, *mexA*, *mexB*, *oprM*, *mexX*, *mexY*, *cpxR*, *nalC*, and *nalD*). All strains possessed all genes, except for *mipA*, which was not detected in strains PA206 and PA216. SNPs were detected in most genes in most strains, with the exceptions of *mexA* (SNP in PA216 only), *aprM* (no SNP in any strain), *cpxR* (SNPs in 6206 only), and *nalD* (SNPs in PA221, PA217, PA220, and PA219 only). Table 5 gives data for genes associated with changes in lipopolysaccharide (LPS) and other factors involved in polymyxin resistance (*oprH*, *papP*, *mpl*, *slyB*, *ppgS*, *ppgH*, *speD2*, *speE2*, *waaL*, *PA5005*, *rsmA*, and *mprF*). Again, most strains possessed SNPs in most genes, with the exceptions of *oprH*, *slyB*, *speD2*, and *rsmA*, which had no SNPs for any strains. Strains were also examined for possession of any of the *mcr* genes that have been shown to be involved in polymyxin resistance, but no strain possessed these genes. All the strains that had their whole genomes available possessed all the genes that had been previously associated with resistance to polymyxins, apart from the *mcr* genes which no strain possessed, and two strains that did not possess *mipA*.

When analyzing associations with polymyxin B MICs, strain PA123, which had the highest MIC of 512 µg/mL, was found to carry a unique combination of SNPs in the following genes: *arnA* (Q661L), *arnB* (Q336L), *mpl* (V358I), *parR* (I93T), *speE2* (A3V), and *waaL* (A110G). Additionally, the *arnT* G156R SNP was shared exclusively between PA123 and strain PA216. PA216, which had an MIC of 256 µg/mL, also harbored *mipB* R401del, *mpl* A303V, and *mprF* N553D variants. The other strains with MIC of 256 μg/mL (PA217, PA219 and PA220) all possessed the SNPs *nalC* E153Q, *nalD* R38W, and *pmrB* V6A, as well as either *mprF* R188H or *mprF* N553D (strain PA219 contained both). Strain PA55, with an MIC of 128 μg/mL only and uniquely possessed *pmrB* H345Y. Strain PA193, the only isolate with an MIC of 8 µg/mL, carried the *waaL* L281F variant, which was also present in strain PA227. Gene *mipB* V469M (*p* = 0.0047) SNP was more common in strains with MIC ≥ 64 but <512 μg/mL than those with MICs of ≤8 μg/mL. Also, strains with MIC ≥ 64 but <512 μg/mL more commonly possessed *mipB* V469M or G441S (*p* = 0.0055) SNPs than those strains with MICs of ≤8 μg/mL.

When examined for associations with colistin resistance, the strain with the highest MIC of 512 μg/mL, PA123, had a unique collection of SNPs in several genes: *arnA* Q661L, *arnB* Q336L, *mpl* V358I, *parR* I93T, *speE2* A3V, and *waaL* A110G (the same as those of this strain in relation to polymyxin resistance). The SNP *arnT* G156R was shared only between strain PA123 and PA216. Strain PA55, with an MIC of 256 μg/mL, only and uniquely possessed *pmrB* H345Y. The other strains with MIC of 256 μg/mL, ATCC 19660 and PA219, also both possessed *mprF* N553D, which was shared with two other strains (PA216 and PA217) with MIC ≥ 64 μg/mL, but no strain with MIC < 64 μg/mL. Strains with MIC ≥ 64 but <512 μg/mL were significantly more likely to possess either *nalC* E153Q/D (*p* = 0.0174), *mipB* V469M (*p* = 0.0174), or *mipB* V469M or G441S (*p* = 0.0007) than strains with MIC ≤ 4 μg/mL.

For Mel4, there were several SNPs that were uniquely possessed by strains with MICs of ≥64 μg/mL compared to strains with MICs of ≤32 μg/mL. These included *arnF* A125T (3 strains), *arnT* A267V (4 strains), *ppgS* N670S (3 strains), *ppgH* R110H (6 strains), *speE2* V217I (3 strains), *mipA* deletion after N34 (3 strains), *waaL* G330A (9 strains), and *PA5005* D23E (3 strains). Strains with MICs of ≥64 μg/mL were more likely to possess *waaL* G330A (*p* = 0.0237) or *mipB* V469M or G441S (*p* = 0.0237).

**Figure 1:**
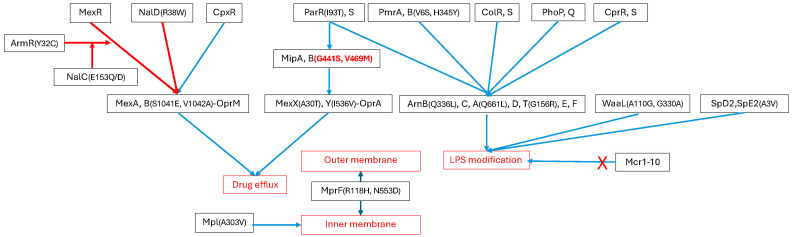
Schematic representation of the genes studied and their effects on cellular processes (red boxes) that result in resistance to polymyxins. Red lines represent inhibition; blues lines represent activation. Red and bold letters and numbers represent amino acid changes to that protein significantly associated with high MICs to polymyxins; other letters and numbers in parentheses represent amino acid changes that may be associated with high MICs in certain strains. The red cross represents the fact that no strain contained any of the *mcr* genes.

For LL-37, several SNPs (or lack thereof) were uniquely possessed by strains with MICs of ≥64 μg/mL compared to strains with MICs of ≤32 μg/mL. These included a lack of SNPs in *pmrA* (6 strains), *pmrB* S2P (7 strains), *arnA* F80Y (4 strains), *arnE* R28H (3 strains), *arnT* A267V (3 strains), *armR* Y32C (5 strains), *mexB* S1041E and V1042A (5 strains), *mexX* A30T (6 strains), *mexY* I536V (6 strains), *nalC* E153Q (6 strains), *nalD* R38W (3 strains), *mipA A32V, S33Q* and *N34-del* (3 strains), and *mipB* V469M (6 strains) or G441S (3 strains). Strains with MICs ≥ 64 μg/mL were significantly more likely to possess *mipB* V469M or G441S (*p* = 0.0075). Figure 1 shows a representation of the genes studied and the amino acid changes that were associated with at least one strain having high MICs to the polymyxins of the AMPs.

## 3. Discussion

This study tested the hypothesis that reduced susceptibility to polymyxins (polymyxin B and colistin) correlates with higher MICs for human cathelicidin LL-37 and the chimeric peptide Mel4, and that these phenotypes would coincide with the presence of, and SNPs within, genes implicated in polymyxin resistance. The hypothesis was at least partially demonstrated, as there were statistically significant correlations between MICs for the polymyxin antibiotics and those for LL-37 and Mel4. Perhaps not surprisingly, given the close chemical similarities between polymyxin B and colistin (polymyxin E), the correlations between MICs for these two antibiotics were the most robust. The moderate correlations may indicate partial overlap of resistance mechanisms, and this was tested in subsequent analyses.

No strain possessed the *mcr* genes. The *mcr* genes have been sporadically reported in *P. aeruginosa*, with only 11.2% of *P. aeruginosa* strains isolated from healthcare-associated infections in Nepal possessing *mcr-1* [34], and only 1% of *P. aeruginosa* clinical isolates from a study in Brazil [35]. The *mcr-5* gene appears to be extremely rare, detected in only 1 out of 2440 *P. aeruginosa* isolates from the U.S. Multidrug-Resistant Organism Repository and Surveillance Network [36]. Another study of 116 carbapenem-resistant *P. aeruginosa* clinical isolates from China also found no evidence for possession of *mcr*-*1–8* or *mcr-10* [14].

Changes to the lipopolysaccharide of *P. aeruginosa* appears to be a relatively common mechanism for resistance to polymyxins. This is often mediated by upregulation of the *arnBCADTEF* operon by several two-component signaling systems such as ParRS, PmrAB, ColRS, PhoPQ, and CprRS [20,21,23], as well as the sensor MipAB [27]. SNPs in these signaling system genes have previously been associated with increased polymyxin resistance.

MipAB is a membrane-associated sensor for polymyxin that is activated by ParRS and upregulates the efflux pump MexXY-OprA [27]. As MipAB has only recently been characterized as being associated with polymyxin resistance, no reports of SNPs affecting its function have been published. The initial report found that some strains of *P. aeruginosa* such as PA14 and PAO1 contained a truncated version of MipA [27] and all strains in the current study either also had truncated *mipA* or did not possess the gene (PA206 and PA216). These differences were not associated with resistance to the polymyxins, LL-37, or Mel4. However, the current study found the *mipB* SNP V469M to be more common in strains with MICs ≥ 64 but <512 μg/mL than those with lower MICs for polymyxin and colistin. Those strains with higher MICs (≥64 μg/mL) for LL-37 or Mel4 were also more likely to possess the SNPs V469M or G441S in *mipB.* This suggests that these SNPs may affect either expression of *mipB,* interaction of MipB with MipA, the interaction with ParRS, or the upregulation of MexXY-OprA, and this should be examined in future studies to determine what the role of these SNPs might be in resistance to the polymyxins, LL-37, and Mel4.

Possession of *pmrA* L71R has been linked to increased expression of this gene in resistant *P. aeruginosa* [14]. However, this SNP was present in nearly all isolates in the current study, including the highly polymyxin-B-susceptible isolate PA189 and the highly resistant isolate PA123, suggesting it is not associated directly with polymyxin resistance in the current strains. Possession of *pmrB* H345Y and *parS* H398R resulted in overexpression of *pmrA* and *arnA* [14]. In the current study, only strain PA55 possessed *pmrB* H345Y, and this strain possessed a unique cohort of SNPs. Its possession of *pmrB* H345Y may be the reason it had high MICs for the polymyxins (128–256 μg/mL), LL-37 (64 μg/mL), and Mel4 (128 μg/mL). No strain in the current study possessed *parS* H398R. Whilst several other specific SNPs in *pmrB* have been linked to increases in colistin resistance [21], none of those SNPs were present in the isolates in the current study, and most *pmrB* SNPs in the current study were not associated with polymyxin B or colistin resistance. Similarly, SNPs in *pmrB* associated with resistance to colistin or the antimicrobial peptide murepavadin [37] were also mostly absent from strains in the current study. The only exception was *pmrB* V6S, which was possessed by one strain with an MIC of 64 μg/mL in the previous study [37] and all strains with an MIC of 256 μg/mL to polymyxin B in the current study, suggesting a link to resistance. A previous study found *pmrB* A247T SNP in two colistin-resistant strains [38]. This SNP was also found in three strains in the current study (PA193, 6206, and PA198) which had MICs ranging from 4 to 64 μg/mL, but was not found in other colistin-resistant strains. Another study found the SNPs *pmrB* V15I and G68S in two colistin-resistant strains [39]. The current study found these SNPs in only one colistin-susceptible strain (PA33) but six colistin-resistant strains (PA217, 6206, PA220, PA221, PA31, and PA219), which, whilst not significantly different, may indicate a role for these SNPs in colistin resistance.

A previous study of highly colistin-resistant (MIC > 512 μg/mL) cystic fibrosis *P. aeruginosa* isolates found various SNPs in *phoQ* to be associated with this resistance [40]. The current study found no SNPs in *phoQ* or *phoP* for any strain, but as all the current MICs were ≤512 μg/mL this may indicate that SNPs in this signaling system are only associated with very high levels of colistin resistance (>512 μg/mL).

The *arnBCADTEF* operon is also regulated by CprRS, which responds to a range of antimicrobial peptides including polymyxin B, CRAMP, and LL-37 [23,41]. Upon stimulation by antimicrobial peptides, CprRS upregulates the HigBA toxin–antitoxin system, which in turn promotes the production of type III secretion system effectors. In the current study, there were several SNPs in *cprR* (E183D) and *cprS* (E111D, A175V, N221H, and T329S) that were shared between four strains (PA225, PA198, PA206, and PA193) but these did not correlate with MICs to the polymyxins, LL-37, or Mel4. These SNPs were not reported in other studies that identified different SNPs in *cprS* [14,38,39]. The same four strains also had SNPs in *parR* (T135A) and *parS* (A115E, V304I, E343D, and Y407H). Again, these did not correlate with MICs to the polymyxins, LL-37, or Mel4. Also, these SNPs were not reported in other studies [14,38,39]. The other two-component signaling system involved in polymyxin resistance, ColRS, had few SNPs. Indeed, there were no SNPs found in *colR* and only three random SNPs in *colS*. This indicates that this system may not be important in polymyxin or antimicrobial peptide resistance in the current collection of strains.

These two-component signaling systems activate the *arnBCADTEF* operon, and the current study found SNPs within genes in this operon. Whilst there were several SNPs within genes in this operon, none appeared to be closely related to MICs to the polymyxins, LL-37, or Mel4. However, the strain with the highest MIC for polymyxin and colistin, PA123, contained a unique set of SNPs (*arnA* Q661L, *arnB* Q336L, *parR* I93T, *mpl* V358I, *speE2* A3V, and *waaL* A110G) along with *arnT* G156R which was shared only with one other strain that also had a high MIC for polymyxin B (PA216). All these SNPs appear to be novel [42] and are worthy of further investigation to determine which mediate higher MICs to the polymyxins.

Efflux pumps can also contribute to polymyxin resistance. As mentioned above, MipAB activates the efflux pump MexXY-OprA [27]. MexXY has also been linked to polymyxin resistance through changes to LPS [43]. MexR is a repressor of *mexAB-oprM* [44]. ArmR is an anti-repressor of MexR and its activity results in expression of the MexAB-OprM efflux pump [44]. The MexAB-OprM efflux pump may mediate colistin tolerance [45]. CpxR activates the MexAB-OprM efflux pump [46]. NalD is also a repressor for the *mexAB-oprM* operon, and NalC represses ArmR expression and thus de-represses efflux pump expression [47]. The current study found various SNPs associated with efflux pumps associated with polymyxin B (*nalC* E153Q and *nalD* R38W), colistin (*nalC* E153Q/D), and LL-37 (*nalC* E153Q, *nalD* R38W, *armR* Y32C, *mexB* S1041E and V1042A, *mexX* A30T, and *mexY* I536V) high MICs. The *nalC* E153Q SNP has been previously found in a strain of *P. aeruginosa* resistant to ciprofloxacin, cefotaxime, and imipenem [48]. The *nalC* E153Q/D SNP has been found in antibiotic-resistant strains of *P. aeruginosa,* with the *nalC* E153Q SNP linked to patient deaths [47]. The other SNPs appear to be novel and so should be examined for their effects on the expression of efflux pumps and the MICs of polymyxin, LL-37, and Mel4.

Another mechanism for polymyxin resistance is changes to the outer membrane that are independent of the *arnBCADTEF* operon. In the current study, the *waaL* G330A SNP was associated with high MICs to Mel4, and *waaL* A110G to high MICs for polymyxin B and colistin. Whilst these SNPs have not been previously associated with resistance, WaaL is a ligase involved in linking the O-antigen polysaccharide to the core of the *P. aeruginosa* lipopolysaccharide [49], and so the SNPs may affect this function. This should be followed up in future research. Other SNPs in *waaL*, Y58F, A354S, L363M, and P364S have been found in a highly polymyxin-resistant *P. aeruginosa* strain [14]. SpE and SpD are involved in spermidine synthesis [50], and spermidine can bind lipopolysaccharide and stabilize and protect the outer membrane of *P. aeruginosa* in response to polymyxin [50]. In the current study, *speE2* A3V was found in strains with high MICs to polymyxin B and colistin but did not appear to be associated with MICs to LL-37 or Mel4. The *mpl* gene has been shown to be involved in maintaining inner membrane integrity in *P. aeruginosa* and has a role in polymyxin resistance [51]. The current study found the *mpl* A303V SNP to be associated with high polymyxin resistance. MprF is an enzyme that modifies anionic phospholipids with L-lysine or L-alanine to give them positive charges into the membrane surface [52]. This can confer resistance to cationic peptides. In *Staphylococcus aureus* the S295L, T345A, L826F, and S829L SNPs in *mprF* have been associated with daptomycin resistance [53,54,55]. These SNPs have also been linked to resistance to cationic host defense peptides [56]. The current study found high polymyxin MICs were linked with *mprF* R188H or N553D SNPs. Whether these function in similar ways to the *S. aureus* SNPs in *mprF* should be examined in future studies.

## 4. Materials and Methods

### 4.1. Bacterial Strains

Clinical isolates of *Pseudomonas aeruginosa*, mostly from cases of microbial keratitis in Australia and India, were randomly selected and retrieved from the microbiology biobank (School of Optometry and Vision Science, UNSW Sydney, Australia), where they had been stored at −80 °C. Isolates were revived in nutrient broth (Oxoid Ltd., Basingstoke, Hampshire, UK) prior to testing.

Table 6 provides details of the 40 *P. aeruginosa* strains used in the current investigation. Strains, randomly selected from the biobank at the School of Optometry and Vision Science, UNSW Sydney, Australia, came from either cases of keratitis (32/40) or cystic fibrosis (4/40) or from other sources. Fifty percent of strains were from Australia; others were from India (16/40) or the USA (1/40) or were of unknown origin. Eighty percent of strains had previously been screened for susceptibility to traditional antibiotics [57,58,59,60,61,62,63,64,65], and the antibiograms are given in Table 6. Whole genome sequences were downloaded, for those that were available, from the NCBI database, and the biosample numbers are given in Table 6.

### 4.2. Antimicrobial Susceptibility Testing

The minimum inhibitory concentrations (MICs) of colistin, polymyxin B, Mel4, and LL-37 against *P. aeruginosa* were determined by the broth microdilution method in cation-adjusted Mueller–Hinton broth (CAMHB, Becton Dickinson and Company, Franklin Lakes, NJ, USA). A standardized bacterial inoculum was prepared by adjusting an overnight culture to a 0.5 McFarland standard (~1.5 × 10^8^ colony forming units (CFU)/mL), followed by further dilution in CAMHB to achieve a final concentration of approximately 1.5 × 10^6^ CFU/mL. Serial two-fold dilutions of the antimicrobial working solutions were prepared across a 96-well microtiter plate, with concentrations ranging from 0.125 to 512 µg/mL. Bacterial growth and sterility controls were included to ensure assay validity. The wells were inoculated with 100 µL of the prepared bacterial suspension and incubated at 37 °C for 18–24 h. MICs were determined by visual inspection and confirmed by measuring their absorbance at 660 nm (OD660) using a FLUOstar Omega microplate reader (BMG LABTECH, Ortenberg, Germany). The MIC was defined as the lowest antibiotic concentration producing 90% growth inhibition relative to the untreated growth control. The reference strain *P. aeruginosa* PA01 was used to validate test accuracy, ensuring reproducibility and alignment with the clinical and Laboratory Standards Institute (CLSI) and European Committee on Antimicrobial Susceptibility Testing (EUCAST) guidelines.

### 4.3. Detection of Polymyxin Resistance Genes and SNPs

The available whole genome sequences of strains (21 in total; Table 6) were downloaded from the NCBI database from bioprojects PRJNA590804 and PRJNA431326, as well as by searching for individual sequences (ATCC 19660 [biosample SAMN01918025] and 6206 [biosample SAMN12437401]), and details of the genomes can be found in the NCBI database and the original publications [58,65]. Genes in these sequences were compared to the sequence of polymyxin-resistance genes in *P. aeruginosa* PA01 (accession number NC_002516.2) and other strains (Supplementary Table S1) also downloaded from the NCBI database in BLAST (version 2.17.0) searches. Single nucleotide polymorphism (SNP) calling using BLASTn (version 2.17.0) and amino acid sequences were compared, after translation of the gene sequence using the Expasy Translate tool (https://web.expasy.org/translate/, accessed on 14 September 2025), to protein sequences in the Pseudomonas genome database (https://pseudomonas.com, accessed on 14 September 2025) (or if not present there, from NCBI) using the SIM—Alignment Tool for Protein Sequences in Expasy (https://web.expasy.org/sim/, accessed on 14 September 2025). The genes to be examined were selected from published studies demonstrating them to have associations with polymyxin or AMP resistance, including *mcr* genes [3,14,20,21,23,27,32,37,38,39,40,41,42,43,44,45,46,47,49,50,51,52,56,68,69].

### 4.4. Statistical Analysis

The MIC data was tested for normality of its distribution using the Kolmogorov–Smirnov test; as the data were found to not be normally distributed (*p* < 0.025), assessment of correlations between MICs of the compounds was performed using the Spearman rank test. Fisher’s exact test was used to determine whether there were associations between two categorical variables, MIC and SNP. The null hypothesis was that the SNP distribution was independent of the MIC. As this was the first time, as far as the authors are aware, that such analyses have been conducted with *P. aeruginosa* strains mostly isolated from ocular infections, corrections for multiple testing were not used in order to give directions for future in depth evaluations.

## 5. Conclusions

In conclusion, the current study found correlations between high MICs to polymyxin antibiotics, the human cathelicidin LL-37, and a chimeric antimicrobial peptide Mel4. Analysis of genes associated with polymyxin resistance in the *P. aeruginosa* found several SNPs that could be associated with the cross-resistance. The most significant of these were SNPs in *mipB*, particularly V469M or G441S. The functional effect of these SNPs on MipB should be investigated in future studies. Also, increasing the number of strains examined will help to make the results more generalizable. As no corrections for multiple associations were made in the current study, some of the associations may have occurred by chance, and so focused research on these initial significant associations should be undertaken in future studies.

This may be the first study focused on ocular *P. aeruginosa* isolates showing that elevated polymyxin MICs are moderately but consistently associated with increased MICs of both a host (LL-37) and a synthetic (Mel4) AMP, while suggesting specific SNPs in genes, such as mipB V469M and G441S, as candidate genomic markers of cross-resistance. Prior studies typically examined non-ocular isolates or single AMPs or did not analyze phenotype–genotype associations. Whilst polymyxin antibiotics are not currently used to treat *P. aeruginosa* ocular infections, this may change in the future due to increasing reports of strains being resistant to commonly used antibiotics such as fluoroquinolones, aminoglycosides, and cephalosporins [62,63,64,65]. Polymyxins are increasingly used to treat other infections caused by antibiotic-resistant *P. aeruginosa* [70]. The current results indicate a potential risk as polymyxin resistance in these isolates was surprisingly high. Also, the findings may have implications for the development of AMPs to produce antimicrobial contact lens coatings [33], where the cross-resistance might allow strains to colonize these types of lenses. This should be followed up in future studies. Confirmatory studies of the associations found in the current study, focused on, for example, *mipB* V469M or G441S in separate strains, as well as mutating the parent strain PAO1 with these SNPs to determine the effect on resistance, are needed.

## Figures and Tables

**Table 1:** Minimum inhibitory concentrations of polymyxin B, colistin, Mel4, and LL37 for the *P. aeruginosa* isolates.

Strains	Minimum Inhibitory Concentrations (µg/mL)			
	Polymyxin B	Colistin	Mel4	LL37
PA01—resistant	128	128	128	64
PA01—sensitive	0.5	0.5	0.5	0.5
PA189	0.5	4	64	32
PA122	1	1	32	64
PA9	1	1	32	16
PA179	1	4	32	8
PA214	1	4	32	32
PA33	2	2	16	32
PA182	2	2	16	16
PA223	2	2	32	32
PA224	2	2	128	32
PA212	2	8	256	32
PA213	2	8	32	16
PA206	2	16	16	16
PA196	4	2	128	64
PA126	4	4	16	16
PA31	4	128	128	256
PA193	8	4	32	16
PA209	16	8	32	256
PA124	16	16	32	128
PA229	64	128	32	32
PA225	64	128	64	128
PA226	64	128	64	128
6206	64	128	128	512
PA222	128	64	32	256
PA198	128	64	128	128
PA227	128	64	256	256
PA221	128	128	128	64
ATCC 19660	128	256	64	256
PA55	128	256	128	64
PA56	256	4	32	16
PA65	256	8	64	32
PA217	256	64	16	256
PA216	256	128	64	256
PA220	256	128	256	32
PA54	256	256	32	16
PA219	256	256	128	256
PA83	256	256	256	32
PA123	512	512	16	64

**Table 2:** Correlations between MICs for polymyxin B, colistin, Mel4, and LL37.

Statistical Parameters	Polymyxin B vs. Colistin	Polymyxin B vs. Mel4	Colistin vs. Mel4	Polymyxin B vs. LL37	Colistin vs. LL-37	Mel4 vs. LL-37
$r_{s}$	0.747	0.296	0.389	0.441	0.506	0.419
$r_{s}$ ^2^	0.558	0.088	0.151	0.195	0.256	0.176
*p* value	<0.001	0.063	0.013	0.005	0.001	0.007

**Table 3:** Possession of genes associated with the *arnBCADTEF* operon and its regulation.

Strains	*pmrA*	*pmrB*	*phoP*	*phoQ*	*cprR*	*cprS*	*parR*	*parS*	*colR*	*colS*	*arnA*	*arnB*	*arnC*	*arnD*	*arnE*	*arnF*	*arnT*
PA189	L71R	+	+	+	+	E386D, L411M	+	H398R	+	+	C312S, S313G	V302A, E376D	+	+	+	L114F	V20A, I509V
PA33	+	S2P, A4T, V15I, G68S,	+	+	+	T16S	M59I, L153R, S170N	H398R	+	+	F80Y, C312S, S313G, I388V	V302A, E376D	+	+	+	+	H151Y, A267S, L337Q, T443A, I509V
PA182	L71R	+	+	+	+	E386D, L411M	+	H398R	+	+	C312S, S313G	V302A	+	+	+	V14M	A267S, R445H, I509V
PA223	+	+	+	+	+	+	+	H398R	+	+	C312S, S313G	V302A, E376D	+	+	+	+	C7W, H151Y, M274I, T443A, I509V
PA224	L71R	S2P, A4T	*+*	+	+	+	+	H398R	+	+	+	V302A	+	E25D, F58L, G208S	+	V14M, A125T	S257R, R502Q, I509V, R521H
PA206	D61E, L71R	A4T, P369A, A427T	*+*	+	E183D	E111D, A175V, N211H, T329S, E386D	T135A, L153R	A115E, V304I, E343D, H398R, Y407H	+	H353R	T42I, P57A, I138V, S313G, I388V, T636A	V302A, E376D	E35G, I309V, T316A	E25D, F58L	A109V	V14M	C7W, G14A, V89A, A116T, L163F, T166S, A214V, A404G, T443A, R502Q, I509V, S517G
PA126	V34L, L71R	+	*+*	+	+	+	+	H398R	+	+	C312S, S313G, I388V, L591M	K286E, V302A	+	+	+	+	V266I, T443A, I509V
PA31	+	S2P, A4T, V15I, G68S,	*+*	+	+	T16S	M59I, L153R, S170N	H398R	+	+	F80Y, C312S, S313G, I388V	V302A, E376D	+	F58L	+	+	H151Y, A267V, L337Q, T443A, I509V
PA193	D61E, L71R	A4T, P369A, A427T	*+*	+	E183D	E111D, A175V, N211H, T329S, E386D	T135A, L153R	A115E, V304I, E343D, H398R, Y407H	+	+	T42I, P57A, I138V, S313G, I388V, T636A	V302A, E376D	+	F58L	+	+	D154E, V290L
PA225	D61E, L71R	A4T, P369A, A427T	*+*	+	E183D	E111D, A175V, N211H, T329S, E386D	T135A, L153R	A115E, V304I, E343D, H398R, Y407H	+	+	T42I, P57A, I138V, S313G, I388V, T636A	A175V, V302A	+	G206C	+	V14M, A125T	R445H, R502Q, I509V
6206	+	S2P, A4T, V6A, V15I, G68S,	*+*	+	+	T16S, E386D	L153R, S170N	H398R	+	G285S	C312S, S313G, I388V	K286E, V302A, E376D	L254F	+	R28H	V14M	H151Y, T166I, A267V, I509V
PA198	D61E, L71R	A4T, P369A, A427T	*+*	+	E183D	E111D, A175V, N211H, D220E, T329S, E386D	T135A, L153R	A115E, V304I, E343D, H398R, Y407H	+	+	T42I, P57A, I138V, S313G, I388V, T636A	V302A, E376D	+	+	+	+	H151Y, A267S, L337Q, T443A, I509V
PA227	T31I	+	*+*	+	+	+	+	H398R	+	+	A170T, C312S, S313G, I388V	A175V, V302A	+	G206C	+	V14M, A125T	R445H, R502Q, I509V
PA221	+	S2P, A4T, V6A, V15I, G68S,	*+*	+	+	T16S, E386D	L153R, S170N	H398R	+	+	F80Y, C312S, S313G, I388V	V302A	+	+	R28H	V14M	H151Y, T166I, A267V, I509V
ATCC 19660	L71R	S2P, A4T	*+*	+	+	T16S, A88V, D153N, V159I, E386D	L153R, S170N	H398R	+	A316T	I138V, S313G, I388V, V564I	G72S, K286E, V302A, E376D	+	E25D, F58L, V123A	A109V	V14M	C7W, A214V, A225V, L337Q, I509V
PA55	+	H345Y	*+*	+	+	+	+	+	+	+	+	+	+	+	+	+	+
PA217	+	S2P, A4T, V6A, V15I, G68S,	*+*	+	+	T16S, D386E	L153R, S170N	H398R	+	+	F80Y, C312S, S313G, I388V	Q23L, K286E, V302A, E376D	+	F58L	R28H	+	C7W, H151Y, L337Q, T443A, I509V
PA216	L71R	+	*+*	+	+	+	+	H398R	+	+	C312S, S313G	A259T, V302A, A316V, R340H	+	+	+	+	G156R, A267S
PA220	L71R	S2P, A4T, V6A, V15I, G68S,	*+*	+	+	T16S, E386D	L153R, S170N	H398R	+	+	F80Y, C312S, S313G, I388V	V302A	+	+	R28H	V14M	H151Y, T166I, A265V, G338E, I509V
PA219	+	S2P, A4T, V6A, V15I, G68S,	*+*	+	+	T16S, E386D	L153R, S170N	H398R	+	+	F80Y, C312S, S313G, I388V	V302A, E376D	+	F58L	+	+	H151Y, A267S, L337Q, T443A, I509V
PA123	L71R	S2P, A4T	*+*	+	+	+	I93T	H398R	+	+	C312S, S313G, Q661L	V302A, Q336L	+	+	+	+	G156R, A267S, R502Q, I509V

+ = possession of gene but no SNPs found. Capital letters refer to standard single letter amino acid designations, and the numbers between the letters refer to the position in the amino acid sequence where the change has occurred. ins = insertion of one or more amino acids at that position. del = deletion of amino acids after that position.

**Table 4:** Possession of genes associated with efflux pumps and their regulation.

Strains	*mipA*	*mipB*	*armR*	*mexR*	*mexA*	*mexB*	*oprM*	*mexX*	*mexY*	*cpxR*	*nalC*	*nalD*
PA189	K2R, T3S, A4P, G14S, T20V, Y26D, L28I, L30P, G31S, A32-del	N171S, H195Y, G152_R153insGG, A157_S158insAA, E406K, T413N	+	E126V, V132A	+	T90I	+	K329Q, W358R	A254G, Q282R, T543A	+	G71E, S209R	+
PA33	K2R, T3S, A4P, G14S, T20V, Y26D, L28I, A32-del	D225E, P328L, I350V, S368R, K387R, E406K, I427V, R441S, D494G, T496A, S507P	T13I, S21T, Y32C	E126V	+	T90I, S1041E, V1042A	+	A30T, K329Q, L331V, W358R	I536V, T543A, G589A, Q840E	+	G71E, D79E, S209R	+
PA182	K2R, T3S, A4P, G14S, T20V, Y26D, L28I, A32-del	S8P, H82Q, N171S, H195Y, G152_R153insGG, A157_S158insAA, E406K, I427V, D494G, T496A, S507P, G525S	+	E126V	+	T90I	+	K329Q, L331V, W358R	T543A	+	G71E, S209R	+
PA223	K2R, T3S, A4P, G14S, T20V, A21S, Y26D, L28I, L30P, G31S, S33-del	E40K, N171S, H195Y, H246Q, G255S, A258_S259insAA, I350V, K387R, E406K, P410S, I427V, D494G, T496A, S507P, G525S	+	+	+	T90I	+	K329Q, L331V, W358R	T543A	+	G71E, S209R	+
PA224	K2R, T3S, A4P, G14S, T20V, Y26D, L28I, L30P, G31S, S33-del	N171S, H195Y, G152_R153insGG, A157_S158insAA, P343R, E406K	+	E126V	+	T90I	+	K329Q, L331V, W358R	T543A, Q840E	+	G71E, S209R, P210L	+
PA206	no significant homology	N171S, H195Y, G250S, G58_L59insT, V263T, A341T, K387R, R401P, A402T, E406K, D412E, I427V, F440I, M457A, D494G, T496V, S507P, G550E, T551A, V567I, Q595H, P602H, W603X	T5A, S21T	A103G, E126V	+	T90I, N248K	+	L12P, A30T, V322L, K329Q, L331V, G344D, W358R	T543A, Q840E, G1035D, N1036T, Q1039R, I1040T	+	G71E, Q182K, Q208A, S209D, P210-, A211-, Q212-, G213-	+
PA126	K2R, T3S, A4P, G14S, T20V, Y26D, L28I, L30P, G31S, A32-del	N171S, H195Y, P343R, R401del, E456K	+	+	K289R	T90I	+	K329Q, L331V, W358R	T543A	+	G71E	+
PA31	K2R, T3S, A4P, G14S, T20V, Y26D, L28I, A32-del	S8P, H195Y, D125E, P328L, I350V, S368R, K387R, E406K, I427V, G441S, D494G, T496V, S507P	T13I, S21T, Y32C	E126V	+	T90I, S1041E, V1042A	+	A30T, K329Q, L331V, W358R	I536V, T543A, G589A	+	G71E, E153Q, S209R	+
PA193	K2R, T3S, A4P, G14S, T20V, Y26D, L28I, L30P, G31S, S33-del	D494G, T496A, S507P	T5A, S21T	A103G, E126V	+	T90I, N248K	+	K329Q, L331V, W358R	Q282R, T543A, V980I	+	G71E, Q182K, Q208A, S209D, P210-, A211-, Q212-, G213-	+
PA225	K2R, T3S, A4P, G14S, T20V, Y26D, L28I, A32V, S33Q, N34-del	S8P, S368R, K287R, E406K, I427V, Y430H, V469M, D494Q, T496A, S507P	T5A, S21T	A103G, E126V	+	T90I, N248K	+	K329Q, W358R	T543A	+	G71E, Q182K, Q208A, S209D, P210-, A211-, Q212-, G213-	+
6206	K2R, T3S, A4P, G14S, T20V, Y26D, L28I, A32-del	S8P, A28V, H195Y, D225E, I350V, K387R, E406K, I427V, V469M, D494G, T496A, S507P	S21T, Y32C	E126V	+	T90I	+	A30T, K329Q, L331V, W358R	I536V, T543A	S80A, L92R	G71E, E153Q, S209R	+
PA198	K2R, T3S, A4P, G14S, T20V, Y26D, L28I, A32-del	S8P, H195Y, D125E, P328L, I350V, S368R, K387R, E406K, I427V, G441S, D494G, T496A, S507P	+	A103G, E126V	+	T90I, N248K	+	A30T, K329Q, L331V, W358R	I536V, T543A, G589A, Q840E, N1036T	+	G71E, Q182K, Q208A, S209D, P210-, A211-, Q212-, G213-	+
PA227	K2R, T3S, A4P, G14S, T20V, Y26D, L28I, A32V, S33Q, N34-del	S8P, S368R, K287R, E406K, I427V, Y430H, V469M, D494Q, T496A, S507P	+	+	+	T90I	+	K329Q, W358R	T543A	+	G71E, E153D, A186T	+
PA221	K2R, T3S, A4P, G14S, T20V, Y26D, L28I, A32-del	S8P, H195Y, D125E, I350V, K387R, E406K, I427V, V469M, D494G, T496A, S507P	S21T, Y32C	E126V	+	T90I, S1041E, V1042A	+	A30T, K329Q, L331V, W358R	I536V, T543A	+	G71E, E153Q, S209R	R38W
ATCC 19660	K2R, T3S, A4P, G14S, T20V, Y26D, L28I, A32V, S33Q, N34-del	S8P, A28V, F47L, H95Y, D225E, Q228M, G255S, R288H, I350V, K387R, E406K, R423H, I427V, V469M, D494G, T496A, S507P, G524S	S21T, G23E, Y32C	E126V	+	T90I, I186V, S1041E, V1042A	+	A30T, K329Q, L331V, W358R	I536V, T543A, Q840E	+	G71E, A145V, S209R	+
PA55	K2R, T3S, A4P, G14S, T20V, Y26D, L28I, L30P, G31S, S33-del	+	+	+	+	T90I	+	+	+	+	+	+
PA217	K2R, T3S, A4P, G14S, T20V, Y26D, L28I, S33-del	S8P, A28V, D225E, A258_S259insAA, I350V, R356H, S368R, K387R, E406K, V469M, D494G, T496A, S507P	+	E126V	+	T90I, S1041E, V1042A	+	A30T, K329Q, L331V, W358R	T543A	+	G71E, E153Q, S209R	R38W
PA216	no significant homology	N180S, H195Y, P343R, R401del, E406K	+	+	+	T90I	+	K329Q, L331V, W358R	T543A	+	G71E, A186T	+
PA220	K2R, T3S, A4P, G14S, T20V, Y26D, L28I, A32-del	S8P, A28V, H195Y, D225E, I350V, K387R, E406K, I427V, V469M, D494G, T496A, S507P	S21T, Y32C	E126V	+	T90I, S1041E, V1042A	+	A30T, K329Q, L331V, W358R	I536V, T543A	+	G71E, E153Q, S209R	R38W
PA219	K2R, T3S, A4P, G14S, T20V, Y26D, L28I, A32-del	S8P, H195Y, D225E, P328L, I350V, S368R, K387R, E406K, I427V, G441S, D494G, T496A, S507P	T13I, S21T, Y32C	E126V	+	T90I, S1041E, V1042A	+	A30T, K329Q, L331V, W358R	I536V, T543A, N1036T	+	G71E, E153Q, S209R	R38W
PA123	K2R, T3S, A4P, G14S, T20V, Y26D, L28I, L30P, G31S, A32-del	N171S, H195Y, H246Q, A258_S259insAA	+	+	+	T90I	+	K329Q, L331V, W358R	T543A, Q840E	+	G71E, S209R	+

+ = possession of gene but no SNPs found. Capital letters refer to standard single letter amino acid designations, and the numbers between the letters refer to the position in the amino acid sequence where the change has occurred. ins = insertion of one or more amino acids at that position. del = deletion of amino acids after that position.

**Table 5:** Possession of genes associated with changes to lipopolysaccharide and other factors related to polymyxin resistance in *P. aeruginosa*.

Strains	*oprH*	*papP*	*mpl*	*slyB*	*ppgS*	*ppgH*	*speD2*	*speE2*	*waaL*	*PA5005*	*rsmA*	*mprF*
PA189	+	M18L, S56G, M62L	M297V	+	D553N	A40D, R110H, A165S, K209Q, Q230K, P388S	+	+	S8T, T93A, R147Q	H399N	+	S81G, A748V
PA33	+	M18L, M62L	+	+	G81S, H187R, D553N, Q792K	A40D, A50V, K209Q, P388S	+	T271A	S8T, T93A, R147Q, L179F, G330A, I398T	+	+	R187H, N554D, N670S, A731T, A748V, D772E, K793Q
PA182	+	M18L, S56G, M62L	+	+	G81S, H187R, L199S, D553N, G587S, A731T, E772D	A40D, A165S, K209Q, P388S	+	+	S8T, R147Q	+	+	G587S, A731T, A748V, K793Q
PA223	+	M18L, S56G, M62L	+	+	G81S, H187R, L199S, D553N, G587S	A40D, G95S, A165S, K209Q, Q230K	+	+	S8T, Y58F, L61I, R64Q, G71R, F75L, F78I, S83A, S90L, T98A, L102F, A109V, F112L, A115V, A116G, E121Q, L124E, K127R, T128N, A129I, I139F, S140A, A143V, L145V, L146V, R147H, Y149H, W150L, D151Q, A152T, N153H, P154_L156insAW, L155M, T158S, A178V, L179V, A182V, P189H, I190L, I197L, L201I, G203C, G204C, I207L, A208S, V216I, G217A, A221C, M223G, V226L, L227V, D230N, R231Q, A234T, A237V, L238I, A239G, L242A, A243L, A245M, L246I, L247V, G248A, L251F, Y252N, V255L, I256V, A261V, A269S, D270E, A271S, S276G, V292K, S294A, I316V, V322S, G330V, S332A, K337R, S338D, A340M, A354S, L363M, P364S, M377L, I385V, Q387K	H399N	+	G587S, A748V, D772E, K793Q
PA224	+	M18L, S56G, M62L	M297V	+	G81S, H187R, L199S, D553N, G587S, A731T, E772D	A40D, A165S, K209Q, Q230K, P388S	+	+	S8T, T93A, R147Q	H399N	+	G587S, A731T, A748V, K793Q
PA206	+	M18L, S56G, M62L, S74G	M297V	+	D70E, V103I, H187R, V192I, A545V, G587S, A731E	A40D, A109T, S190R, S203T, K209Q, Q230K	+	T271A	S8T, Y58F, L61I, R64Q, G71R, F75L, F78I, S83A, S90L, T98A, L102F, A109V, F112L, A115V, A116G, E121Q, L124E, K127R, T128N, A129I, I139F, S140A, A143V, L145V, L146V, R147H, Y149H, W150L, D151Q, A152T, N153H, P154_L156insAW, L155M, T158S, A178V, L179V, A182V, P189H, I190L, I197L, L201I, G203C, G204C, I207L, A208S, V216I, G217A, A221C, M223G, V226L, L227V, A228V, D230N, R231Q, A234T, A237V, L238I, A239G, L242A, A243L, A245M, L246I, L247A, G248A, L251F, Y252N, V255L, I256V, A261V, A269S, D270E, A271S, S276G, V292K, S294A, I316V, V322S, G330V, S332A, K337R, S338D, A340M, A354S, L363M, P364S, M377L, I385V, Q387K	Q396K, H399N, R403Q	+	D71E, S82G, V104I, V93I, S100L, V546A, N554D, G587S, A731E, A748V, D772E, K793Q
PA126	+	M18L, S56G, M62L	+	+	G81S, D175N, D553N, G587S	A40D, G95S, A165S, K209Q	+	+	S8T, T93A, R147Q	H399N	+	D175N, R187H, S199L, G587S, A748V, D772E, K793Q
PA31	+	M18L, M62L	D411A	+	G81S, L199S, N670S, A731T	A40D, A50V, K209Q, P388S	+	V217I, T271A	S8T, T93A, R147Q, L179F, G330A, I398T	H399N	+	R187H, N554D, N670S, A731T, A748V, D772E, K793Q
PA193	+	S56G, M62L	M297V	+	H65R, H187R, L199S, G304S, D553N, E772D, Q792K	A40D, R110H, A165S, K209Q, Q230K, P388S	+	+	S8T, R147Q, L281F	H399N	+	H65R, S81G, G304S, A758V
PA225	+	S56G, M62L	M297V	+	H187R, L199S, D553N, I740M, E772D	A40D, R110H, A165S, K209Q, Q230K, P388S	+	+	S8T, T93A, R147Q, L179F, S276G, G330A	H399N	+	S81G, I740M, A748V, K793Q
6206	+	M18L, M62L	+	+	P14L, G81S, L199S, D553N, G587S	A40D, R110H, A165S, K209Q, P231A, P388S	+	+	S8T, T93A, A129V, R147Q, L179F, I256F, S276G, G330A	D23E, H399D	+	P13L, R187H, G587S, A748V, D772E, K793Q
PA198	+	M18L, M62L	+	+	G81S, L199S, N670S, A731T	A40D, A50V, K209Q, P388S	+	V217I, T271A	S8T, T93A, R147Q, L179F, G330A, I398T	H399N	+	R187H, N554D, N670S, A731T, A748V, D772E, K793Q
PA227	+	M18L, S56G, M62L	M297V, A303V	+	H187R, L199S, D553N, I740M, E772D	A40D, R110H, A165S, K209Q, Q230K, P388S	+	+	S8T, R147Q, L281F, S276G, G330A	H399N	+	S81G, I740M, A748V, K793Q
PA221	+	M18L, M62L	+	+	G81S, L199S, D553N, G587S	A40D, R110H, A165S, K209Q, P231A, P388S	+	+	S8T, T93A, A129V, R147Q, L179F, I256F, S276G, G330A	D23E, H399D	+	R187H, G587S, A748V, D772E, K793Q
ATCC 19660	+	+	+	+	E616G, A731Y	K209Q	+	+	S8T, T93A, R147Q, L179F, G330A	H399N	+	S81G, R187H, S199L, N553D, E616G, A731T, A748V, D772E, K793Q
PA55	+	M18L, S56G, M62L	+	+	G81S, H187R, L199S, D553N, G587S, E772D	A40D, A165S, K209Q, P388S	+	+	+	+	+	G587S, A748V, K793Q
PA217	+	M18L, M62L	+	+	L199S, A270V	A40D, K209Q, P388S	+	C161S, A165T, V217T, S244A, T271A, E277D, S244A, T271A, E277D, P326A, E235G	S8T, R16H, Y58F, L61I, R64Q, G71R, F75L, F78I, S83A, S90L, T98A, L102F, A109V, F112L, A115V, A116G, E121Q, L124E, K127R, T128N, A129I, I139F, S140A, A143V, L145V, L146V, R147H, Y149H, W150L, D151Q, A152T, N153H, P154_L156insAW, L155M, T158S, A178V, L179V, A182V, P189H, I190L, I197L, L201I, G203C, G204C, I207L, A208S, V216I, G217A, A221C, M223G, V226L, L227V, D230N, R231Q, A234T, A237V, L238I, A239G, L242A, A243L, A245M, L246I, L247V, G248A, L251F, Y252N, V255L, I256V, A261V, A269S, D270E, A271S, S276G, V292K, S294A, I316V, V322S, G330I, S332A, K337R, S338D, A340M, A354S, L363M, P364S, M377L, I385V, Q387K	H399N, N400D	+	S81G, R187H, A270V, N553D, A748V, D772E, K793Q
PA216	+	M18L, S56G, M62L	M297V, A303V	+	A12T, G81S, H187R, L199S, L243M	A40D, K209Q	+	+	S8T, Y58F, L61I, G71R, F75L, F78I, S83A, S90L, T98A, L102F, A109V, F112L, A116G, E121Q, L124E, K127R, T128A, A129L, V138T, I139L, S140A, A143V, R147H, Y148F, Y149H, W150I, D151Q, A152S, N153P, P154_L156insAW, L155M, T158S, A178V, A182V, P189H, I190A, I197L, L201I, G203C, G204C, I207L, V216I, T219A, A221C, L222M, M223A, V226L, A234T, A237V, L238I, A239G, L240I, A241V, L242V, A245L, L247V, G248V, L251F, L252V, Y253N, V255L, T257I, A261V, A269S, D270E, A271S, S276G, V292K, S294A, I316V, V322S, G330V, S332A, K337R, S338D, A340M, A354S, L363M, P364S, M377L, I385V, Q387K	H399N	+	A12T, L242M, N553D, A731T, A748V, D772E, K793Q
PA220	+	M18L, M62L	+	+	G81S, L199S, D553N, G587S	A40D, R110H, A165S, K209Q, P231A, P388S	+	T271A	S8T, T93A, A129V, R147Q, L179F, I256F, S276G, G330A	D23E, H399D	+	R188H, G587S, D772E, K793Q
PA219	+	M18L, M62L	+	+	G81S, L199S, N670S, A731T	A40D, A50V, K209Q, P388S	+	V217I, T271A	S8T, T93A, R147Q, L179F, G330A, I398T	H399N	+	R188H, N553D, N670S, A731T, A748V, D772E, K793Q
PA123	+	M18L, S56G, M62L	M297V, V358I	+	G81S, H187R, L199S, D553N, G587S, E772D	A40D, A165S, K209Q, P388S	+	A3V	S8T, Y58F, L61I, R64Q, G71R, F75L, F78I, S83A, S90L, T98A, L102F, A109V, A110G, F112L, A115V, A116G, E121Q, L124E, K127R, T128N, A129I, I139F, S140A, A143V, L145V, L146V, R147H, Y149H, W150L, D151Q, A152T, N153H, P154_L156insAW, L155M, T158S, A178V, L179V, A182V, P189H, I190L, I197L, L201I, G203C, G204C, I207L, A208S, V216I, G217A, A221C, M223G, V226L, L227V, D230N, R231Q, A234T, A237V, L238I, A239G, L242A, A243L, A245M, L246I, L247V, G248A, L251F, Y252N, V255L, I256V, A261V, A269S, D270E, A271S, S276G, V292K, S294A, I316V, V322S, G330V, S332A, K337R, S338D, A340M, A354S, L363M, P364S, M377L, I385V, Q387K	H399N	+	G587S, A748V, K793Q

+ = possession of gene but no SNPs found. Capital letters refer to standard single letter amino acid designations, and the numbers between the letters refer to the position in the amino acid sequence where the change has occurred. ins = insertion of one or more amino acids at that position. del = deletion of amino acids after that position.

**Table 6:** Details of the Pseudomonas aeruginosa isolates.

Strain Number	Infection Type, Site of Isolation, Country and Date of Isolation	Known Antibiotic Resistance Characteristics	Biosample Number (NCBI)
PA01— resistant	Mutant derived from PAO1-sensitive [57]	Chloramphenicol, Colistin [57]	Not available
PA01— sensitive	Wound, Unknown, Australia, 1954	Chloramphenicol [57,66]	SAMN02603714
PA189	Keratitis, Eye, India, 2017	CIP ^S^, LEV ^S^, GEN ^S^, TOB ^R^, PIP ^S^, IMI ^R^, CFT ^S^ [63]	SAMN13340385
PA122	Keratitis, Eye, Australia, 2006	Not known	Not available
PA9	Keratitis, Eye, Australia, 1994	Not known	Not available
PA179	Keratitis, Eye, Australia, 2006	CIP ^R^, LEV ^S^, GEN ^S^, TOB ^S^, PIP ^S^, IMI ^S^, CFT ^S^ [64]	Not available
PA214	Keratitis, Eye, India, 2017	Not known	Not available
PA33	Keratitis, Eye, India, 1998	CIP ^R^, LEV ^R^, GEN ^R^, TOB ^R^, PIP ^R^, IMI ^R^, CFF ^R^ [63]	SAMN08435058
PA182	Keratitis, Eye, Australia, 2006	CIP ^S^, LEV ^S^, GEN ^S^, TOB ^S^, PIP ^S^, IMI ^R^, CTF ^S^ [63]	SAMN13340383
PA223	Keratitis, Eye, Australia, 2018	CIP ^R^, LEV ^S^, GEN ^S^, TOB ^S^, PIP ^R^, IMI ^S^, CFT ^I^ [63]	SAMN16123412
PA224	Keratitis, Eye, Australia, 2018	CIP ^R^, LEV ^S^, GEN ^S^, TOB ^S^, PIP ^S^, IMI ^R^, CFT ^I^ [63]	SAMN16123413
PA212	Keratitis, Eye, India, 2017	CIP ^R^, LEV ^S^, GEN ^S^, TOB ^S^, PIP ^S^, IMI ^R^, CFT ^S^ [58]	Not available
PA213	Keratitis, Eye, India, 2017	CIP ^R^, LEV ^S^, GEN ^S^, TOB ^S^, PIP ^S^, IMI ^I^, CFT ^I^ [58]	Not available
PA206	Keratitis, Eye, India, 2017	CIP ^I^, LEV ^S^, GEN ^S^, TOB ^S^, PIP ^S^, IMI ^S^, CFT ^S^ [59]	SAMN13340389
PA196	Keratitis, Eye, India, 2017	CIP ^S^, LEV ^S^, GEN ^S^, TOB ^S^, PIP ^S^, IMI ^R^, CFT ^S^ [59]	Not available
PA126	Keratitis, Eye, Australia, 2006	CIP ^S^, LEV ^S^, GEN ^S^, TOB ^S^, PIP ^S^, IMI ^R^, CFT ^R^ [64]	SAMN13340377
PA31	Keratitis, Eye, India, 1998	CIP ^R^, LEV ^R^, MOX ^R^, GEN ^R^, CFT ^I^, CEFE ^I^, IMI ^I^, TIC ^I^, AZT ^I^ [65]	SAMN08435056
PA193	Keratitis, Eye, India, 2017	CIP ^S^, LEV ^S^, GEN ^S^, TOB ^S^, PIP ^S^, IMI ^S^, CFT ^S^ [58]	SAMN13340386
PA209	Keratitis, Eye, India, 2017	CIP ^I^, LEV ^I^, GEN ^S^, TOB ^S^, PIP ^R^, IMI ^S^, CFT ^S^ [63]	Not available
PA124	Keratitis, Eye, Australia, 2006	CIP ^I^, LEV ^S^, GEN ^S^, TOB ^S^, PIP ^S^, IMI ^R^, CFT ^S^ [63]	Not available
PA229	Keratitis, Eye, Australia, 2018	CIP ^S^, LEV ^S^, GEN ^S^, TOB ^S^, PIP ^S^, IMI ^R^, CFT ^S^ [63]	Not available
PA225	Keratitis, Eye, Australia, 2018	CIP ^R^, LEV ^R^, GEN ^S^, TOB ^S^, PIP ^S^, IMI ^R^, CEF ^S^ [63]	SAMN16123414
PA226	Keratitis, Eye, Australia, 2018	CIP ^I^, LEV ^I^, GEN ^S^, TOB ^S^, PIP ^R^, IMI ^R^, CFT ^S^ [63]	Not available
6206	Keratitis, Eye, USA, 1995	CIP ^S^, TOB ^S^ [60,61]	SAMN12437401
PA222	Keratitis, Eye, Australia, 2017	CIP ^S^, LEV ^I^, GEN ^S^, TOB ^S^, PIP ^S^, IMI ^R^, CFT ^R^ [63]	Not available
PA198	Keratitis, Eye, India, 2017	CIP ^R^, LEV ^R^, MOX ^R^ [62]	SAMN13340387
PA227	Keratitis, Eye, Australia, 2018	CIP ^R^, LEV ^R^, GEN ^S^, TOB ^S^, PIP ^S^, IMI ^R^, CFT ^I^ [63]	SAMN16123415
PA221	Keratitis, Eye, India, 2017	CIP ^R^, LEV ^R^, GEN ^R^, TOB ^R^, PIP ^R^, IMI ^R^, CFT ^R^ [63]	SAMN13340395
ATCC 19660	Unknown, 1965	CIP ^I^, GEN ^S^ [61,67]	SAMN01918025
PA55	Cystic Fibrosis, Sputum, Australia, 2003	CIP ^S^, LEV ^S^, MOX ^S^, CFT ^R^, CEFE ^R^, IMI ^R^, TIC ^R^, AZT ^S^ [65]	SAMN08435068
PA56	Cystic Fibrosis, Sputum, Australia, 2003	Not known	Not available
PA65	Cystic Fibrosis, Sputum, Australia, 2003	Not known	Not available
PA217	Keratitis, Eye, India, 2017	CIP ^R^, LEV ^R^, GEN ^S^, TOB ^S^, PIP ^R^, IMI ^R^, CFF ^R^ [63]	SAMN13340391
PA216	Keratitis, Eye, India, 2017	CIP ^R^, LEV ^R^, GEN ^S^, TOB ^S^, PIP ^R^, IMI ^R^, CFT ^R^ [63]	SAMN13340390
PA220	Keratitis, Eye, India, 2017	CIP ^R^, LEV ^S^, GEN ^S^, TOB ^S^, PIP ^S^, IMI ^R^, CFT ^R^ [63]	SAMN13340394
PA54	Keratitis, Eye, Australia, 2003	Not known	Not available
PA219	Keratitis, Eye, India, 2017	CIP ^R^, LEV ^S^, GEN ^S^, TOB ^S^, PIP ^S^, IMI ^R^, CFT ^I^ [63]	SAMN13340393
PA83	Cystic Fibrosis, Sputum, Australia, 2003	Not known	Not available
PA123	Keratitis, Eye, Australia, 2006	CIP ^I^, LEV ^I^, GEN ^S^, TOB ^S^, PIP ^S^, IMI ^I^, CFT ^I^ [63]	SAMN13340376

CIP = ciprofloxacin, LEVO = levofloxacin, MOX = moxifloxacin, CFT = ceftazidime, CEFE = cefepime, TIC = ticarcillin, IMI = imipenem, AZT = Aztreonam, PIP = piperacillin, TOB = tobramycin, GEN = gentamicin. ^S^ = susceptible, ^R^ = resistant, ^I^ = intermediate.

## Data Availability

The original contributions presented in this study are included in the article/Supplementary Material. Further inquiries can be directed to the corresponding author.

## Supplementary Materials

The following supporting information can be downloaded at: https://www.mdpi.com/article/10.3390/ijms262110499/s1.

## Abbreviations

The following abbreviations are used in this manuscript:

AMP	Antimicrobial peptide
MIC	Minimum inhibitory concentration
CARD	Comprehensive Antibiotic Resistance Database
NCBI	National Center for Biotechnology Information
BLAST	Basic Local Alignment Search Tool
SNP	Single nucleotide polymorphism
MDR	Multidrug resistant
XDR	Extensively drug resistant
LPS	Lipopolysaccharide
L-Ara4N	4-amino-4-deoxy-L-arabinose
