# Supplementary material for: Detection of Genes Associated with Polymyxin and Antimicrobial Peptide Resistance in Isolates of Pseudomonas aeruginosa
Source: Int J Mol Sci. 2025 Oct 29;26(21):10499. doi: 10.3390/ijms262110499 (PMC12607733; doi:10.3390/ijms262110499)
Supplement: Supplementary file 1 [file ijms-26-10499-s001.zip › ijms-3914841-supplementary.pdf]

Supplementary Table S1: Identity of genes studied

Gene	Gene alias	*Pseudomonas* strain	NCBI gene ID
pmrA	PA4776	PAO1	881834
pmrB	PA4777	PAO1	881841
phoP	PA1179	PAO1	879194
phoQ	PA1180	PAO1	879187
cprR	B7D75_RS09685	Cr1	77220414
cprS	B7D75_RS09680	Cr1	77220413
parR	PA1799	PAO1	878354
parS	PA1798	PAO1	878335
colR	NCGM2_1222	NCGM2.S1	12571012
colS	NCGM2_1223	NCGM2.S1	12571013
arnA	PA3554	PAO1	878473
arnB	PA3552	PAO1	879143
arnC	PA3553	PAO1	878472
arnD	PA3555	PAO1	879121
arnE	PA3557	PAO1	879132
arnF	PA3558	PAO1	879133
arnT	PA3556	PAO1	879122
mipA	B7D75_RS16010	Cr1	77221614
mipB	PA1797	PAO1	878296
armR	PA3719	PAO1	880376
mexR	PA0424	PAO1	877857
mexA	PA0425	PAO1	877855
mexB	PA0426	PAO1	877852
oprM	PA0427	PAO1	877851
mexX	B7D75_14880	Cr1	77221395
mexY	B7D75_14885	Cr1	77221396
cpxR	NCGM2_4319	NCGM2.S1	12574109
nalC	PA3721	PAO1	880362
nalD	PA3574	PAO1	880183
oprH	PA1178	PAO1	878005
papP	PA14_11960	PA14	4383498
mpl	PA14_11970	PA14	4383497
slyB	PA14_50740	PA14	4384762
ppgS	PA14_52350	PA14	4383150
ppgH	PA14_52370	PA14	4383149
speD2	PA4773	PAO1	881823

speE2	PA4774	PAO1	881824
waaL	PA4999	PAO1	881661
PA5005		PAO1	881615
rsmA	PA0905	PAO1	878352
mprF	B7D75_20595	Cr1	77222499
